# Expression of the growth hormone secretagogue receptor 1a (GHS‐R1a) in the brain

**DOI:** 10.14814/phy2.15113

**Published:** 2021-11-09

**Authors:** Marat I. Airapetov, Sergei O. Eresko, Andrei A. Lebedev, Evgenii R. Bychkov, Petr D. Shabanov

**Affiliations:** ^1^ Department of Neuropharmacology Institute of Experimental Medicine St. Petersburg Russia; ^2^ Department of Pharmacology St. Petersburg State Pediatric Medical University St. Petersburg Russia; ^3^ Research and Training Center of Molecular and Cellular Technologies St. Petersburg State Chemical Pharmaceutical University St Petersburg Russia; ^4^ Department of Biology Saint‐Petersburg State University St Petersburg Russia; ^5^ Department of Pharmacology Kirov Military Medical Academy St. Petersburg Russia

**Keywords:** brain, ghrelin, GHS‐R1a

## Abstract

The review presents data on the expression of growth hormone secretagogue receptor 1a (GHS‐R1a) in the brain regions in model animals (zebrafish, rodents, primates), and in the human brain. Studies show widespread distribution of the receptor in the brain, which evidences the involvement of the receptor in many physiological processes. Using various organisms, data have been obtained regarding the participation of the GHS‐R1a in the regulation of the anti‐ and pro‐inflammatory response, proliferation, and apoptosis. It is known that the receptor plays an important role in eating behavior and is also involved in the pathogenetic mechanisms of drug addiction, obesity, and chronic alcohol consumption. Based on this, research is underway with the use of various therapeutic agents that can be used for the pharmacological correction of these conditions. This review also presents hypothetical pathways of intracellular signaling, in which GHS‐R1a may participate. A complete understanding of these mechanisms has not yet been reached. The ghrelin intracellular signaling seem to be specific to brain region and, probably, also depend on the metabolic or stress status of the organism.

## Graphical abstract

The review presents data on the expression of GHS‐R1a in the brain in model animals and in the human brain. Every year, new data appear showing that GHS‐R1a plays an important role on physiological processes. Numerous data indicate the involvement of the receptor in the development of neurodegenerative process in various pathological conditions of the brain.

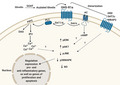

## 1 INTRODUCTION

Since the discovery of the ghrelin receptor (GHS‐R1a, *Growth Hormone Secretagogue Receptor 1a*) more than 20 years have passed, but this receptor remains of great interest. Every year, new data appear showing that GHS‐R1a plays an important role on physiological processes. Much attention is paid to the mechanisms of eating behavior, in which GHS‐R1a is involved, as well as to the contribution of this receptor to various forms of addictive behavior (drug addiction, alcoholism, gambling addiction, etc.) (Akalu et al., 2020; Shabanov et al., 2014; Smith et al., 2012). Numerous data indicate the involvement of the receptor in the development of neurodegenerative process in various pathological conditions of the brain, including Alzheimer's disease and Parkinson's disease (Jeon et al., 2019; Suda et al., 2018). Ghrelin also plays an anti‐neuroinflammatory and neuroprotective role (Buntwal et al., 2019; Shi et al., 2017; Tian et al., 2019). In addition, the participation of ghrelin and its receptor in the mechanisms of stress, leading to behavioral and psychopathological disorders, has been shown (Fritz et al., 2020; Guo et al., 2019).

The aim of this work was to summarize the data on the expression of GHS‐R1a among various structures of the brain obtained on model objects (zebrafish, rodents, primates), as well as on humans. At the moment, such information has not been generalized, which creates difficulties for physiologists when performing research related to the study of the neurophysiology of the ghrelin receptor in the brain.

## 2 GHS‐R1a IN THE BRAIN

### 2.1 Structure and synthesis

The receptor for acylated ghrelin (GHS‐R1a) was first described by Howard et al. (1996). GHS‐R1a is a metabotropic G protein‐coupled receptor (GPCR), these are ubiquitous proteins with seven transmembrane domains that regulate a variety of intracellular signals in response to hormones, neurotransmitters, ions, photons, odorants, and other stimuli. Receptors of this subtype play an important role in cellular processes and are considered an attractive target for drug development. GPCRs are not simple bimodal on/off switches, and should be viewed as highly dynamic systems that exist in many functionally different conformations. Moreover, ligands can regulate the activity of the receptor, changing its conformation and thereby influencing the triggering of intracellular signaling cascades in one direction or another (Hilger et al., 2018).

The rat GHS‐R1a protein consists of 364 amino acid residues that form seven transmembrane domains. Comparison of the complete amino acid sequences of rat, pig, and human GHS‐R1a homologues revealed a high degree of similarity (96.1% in rat and human GHS‐R1a homologues). The GHS‐R gene contains two exons, one of which encodes domains 1–5, and the other, domains 6 and 7. The existence of two types of human and porcine GHS‐R (types 1a and 1b) is explained by alternative splicing of the pre‐mRNA of one gene. The complete GHS‐R1 protein, consisting of seven domains, is encoded by type 1a mRNA, from which an intron located between the two exons of the *GHS*‐*R* gene is removed (McKee et al., 1997).

It is known that acylated ghrelin (AG) serves as a ligand for GHS‐R1a in the central nervous system. AG is formed from the peptide hormone ghrelin as a result of acetylation at the serine residue in position 3 (Ser3) by the enzyme ghrelin‐O‐acyltransferase (GOAT). The presence of an acyl residue is necessary for the binding of ghrelin to its receptor GHS‐R1a in the central nervous system (CNS). There are two known forms of ghrelin: AG and non‐acylated ghrelin, but non‐acylated (des‐acyl ghrelin) does not bind to GHS‐R1a (Ansari et al., 2015). It is assumed that acetylation occurs immediately before the binding of acetylated forms of ghrelin to GHS‐R1a (Abizaid & Hougland, 2019). Des‐acyl ghrelin is the most stable and long‐lived form of ghrelin circulating in blood plasma. The non‐acylated form of ghrelin was originally considered a precursor or a metabolic product of the acylated form, but recently des‐acyl ghrelin has been given a special role in the body (Rauh et al., 2007). The relative content of des‐acyl ghrelin in the total pool of ghrelin is up to 60–90%, while acylated ghrelin is up to 10% of the total ghrelin content (Kempinski et al., 2008). In addition, acylated ghrelin is rapidly degraded in plasma or serum samples, and precise quantification is often associated with some methodological difficulties (Bednarek et al., 2000; Hosoda et al., 2004).

### 2.2 Expression of GHS‐R1a in the brain

The expression of GHS‐R1a was studied in the brain of various model organisms, including zebrafish, mice, rats, primates, and humans.

The aquarium fish zebrafish is a well proven model animal, including in neurobiology. It was found that ghrelin is expressed in zebrafish pancreatic endocrine cells. It was shown using quantitative real‐time RT‐PCR that zGHS‐R1 (a variant with an amino acid sequence slightly different from GHS‐R1a mammals), is expressed at a high level in brain tissue. Expression of GHS‐R1a has also been found in the brains of fish such as tilapia, black sea bream, goldfish, and carp. However, it would be useful to further determine the level of expression of zGHS‐R1 in various parts of the zebrafish brain. (Cai et al., 2015; Eom et al., 2014).

The highest concentrations of GHS‐R1a mRNA are present in the hypothalamus (suprachiasmatic nucleus, ventromedial nucleus, paraventricular nucleus, etc.) and pituitary gland of rats and mice. A high level of ghrelin receptor mRNA was also detected in the dentate gyrus, CA1, CA2, and CA3 of the hippocampus, and in the nuclei of the brain stem (substantia nigra, ventral tegmental region, dorsal raphe nucleus, facial motor nucleus, lateral parabrachial nucleus, and nucleus ambiguus), a small amount of mRNA was found in the piriform cortex of mice and rats. There is data on the expression of GHS‐R1a mRNA in the nuclei of the rat thalamus (Guan et al., 1997; Zigman et al., 2006). mRNA of ghrelin and GHS‐R were also found in the sensorimotor cortex of rats (Hou et al., 2006). Later, it was shown that GHS‐R1a is expressed in several regions of the rat amygdala (Alvarez‐Crespo et al., 2012).

In the guinea pig (*Cavia porcellus*), high levels of GHS‐R1a mRNA expression were found in the pituitary and hypothalamus; moderate levels in the thalamus, cerebral cortex, pons, medulla oblongata, and olfactory bulb; and low levels in the cerebellum and peripheral tissues. The distribution of GHS‐R1a expression in the guinea pig brain was almost the same as in rats. However, the distribution of GHS‐R1a in the peripheral tissues of guinea pigs and rats was different (Kitazawa et al., 2011). French researchers found GHS‐R1a mRNA in the pituitary, hypothalamus, hippocampus, and cerebellar cortex of four adult lemurs (*Microcebus murinus*) (Mitchell et al., 2001).

We found only one work in which the level of GHS‐R1a mRNA was analyzed in humans. GHS‐R1a mRNA was detected in the pituitary, hypothalamus, and hippocampus; however, this work concluded that the inability to detect GHS‐R1a mRNA in other brain regions is most likely due to its low concentration (Guan et al., 1997).

Representation of GHS‐R1a in various structures of the brain (see Table 1), shown using various model objects, indicates the participation of this receptor in many physiological and pathological processes.

**TABLE 1:** Expression of GHS‐R1a in the structures of the brain

Brain structure	Rat	Mouse	Primate	Human
Piriform cortex	+/−	+/−	?	?
Sensorimotor cortex	+	?	?	?
Hippocampus	+	+/−	+	+
Amygdala	+	?	?	?
Hypothalamus	+	+	+	+
Thalamic nuclei	+/−	?	?	?
Midbrain	+	+	?	?
VTA (ventral tegmental area)	+	+	?	+/−
Substantia nigra	+	+	?	+/−
Brainstem nuclei	+	+	?	?
Cerebellum	+	?	+	?
Pituitary	+	+	+	+

Note

“+” ‐ the presence of expression, “+/–” ‐ conflicting data, “?” ‐ no data.

### 2.3 GHS‐R1a and intracellular signaling pathways in the brain

In the brain, GHS‐R1a is localized on the plasma membrane of neurons, astrocytes, and oligodendrocytes (Dong et al., 2019; Lee et al., 2011). Despite the fact that GHS‐R1a is widely expressed by peripheral immune cells (monocytes, macrophages, T cells), the expression of GHS‐R1a was not detected on resident macrophages of the nervous system (microglia) (Bulgarelli et al., 2009; Baatar et al., 2011; Moon et al., 2009).

**FIGURE 1:**
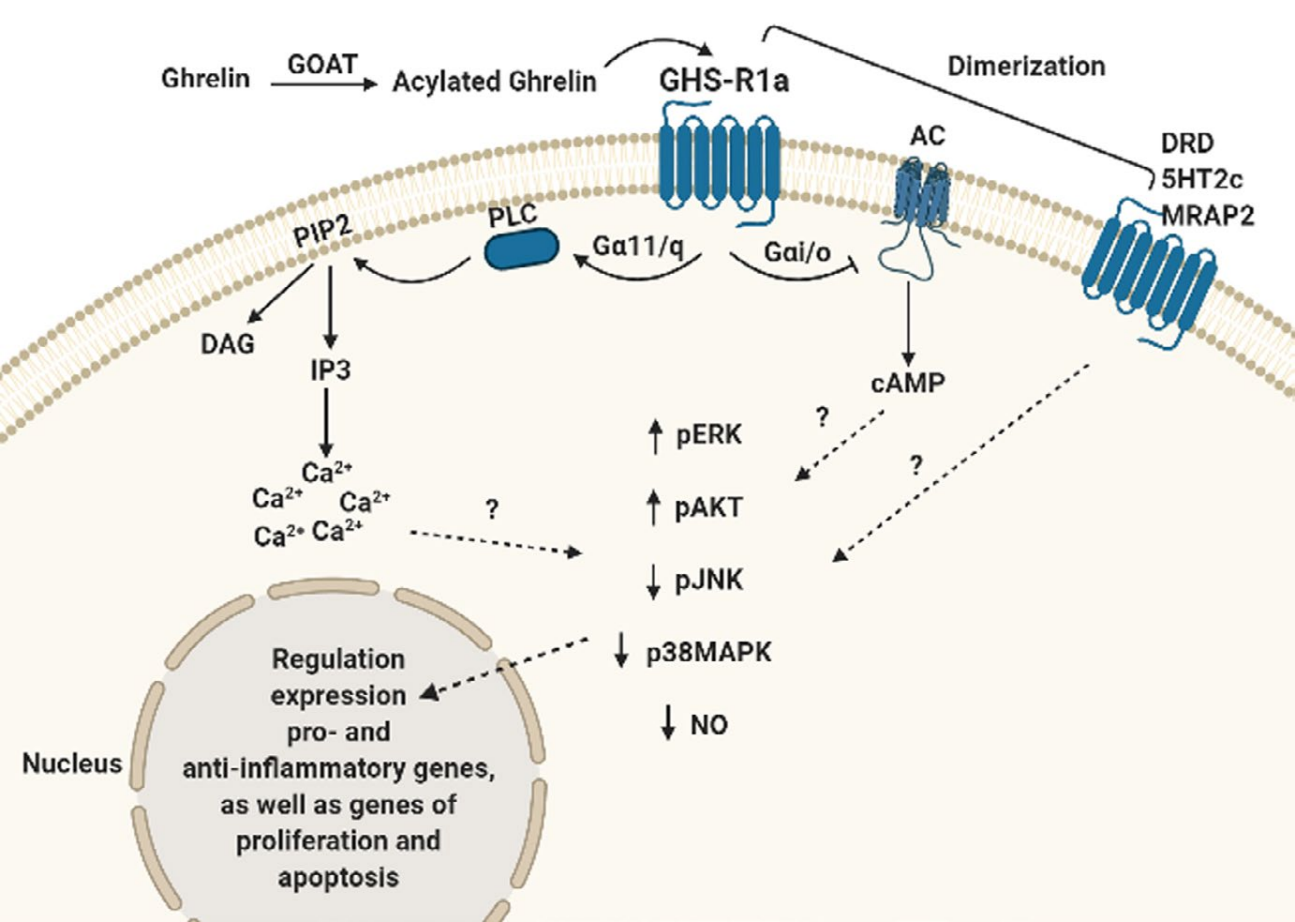
Signaling cascades of GHS‐R1a in brain cells. GOAT, ghrelin‐O‐acyltransferase; AC, adenylate cyclase; PLC, phospholipase C; DAG, diacylglycerol; IP3, inositol‐3‐phosphate; PIP2, phosphatidylinositol diphosphate; DRD, dopamine receptor; 5HT2c, serotonin receptor; MRAP2, melanocortin receptor accessory protein 2

Using GHS‐R1a agonists and antagonists, it was shown that receptor activation leads to changes in many cascades of intracellular reactions (see Figure 1). These intracellular pathways appear to be specific for a certain region of the brain and probably depend on the metabolic or stress status of the individual, as well as on the microenvironment, which contains various signaling molecules (Guo et al., 2019; Song et al., 2018).

The interaction of GHS‐R1a and a ligand activates the Gα11/q signaling pathway–– phospholipase C, which leads to the hydrolysis of phosphatidylinositol diphosphate (PIP2), and consequently to the release of inositol‐3‐phosphate (IP3) and diacylglycerol (DAG). Moreover, GHS‐R1a is also associated with the Gαi/o signaling pathway. Various models have shown that activation of GHS‐R1a leads to an increase in the level of phosphorylation of ERK and AKT kinases, while the level of phosphorylation of JNK and p38MAPK predominantly decreases (Abizaid & Hougland, 2019; Akalu et al., 2020; Lee et al., 2011; Shi et al., 2017; Zeng et al., 2018; Zheng et al., 2019). Activation of ghrelin signaling leads to a decrease in the level of nitric oxide (Zeng et al., 2018). A change in the level of phosphorylation of various protein kinases also leads to a change in the activity of various transcription factors that regulate the expression of a wide variety of genes; ghrelin signaling is involved in changes in the expression level of pro‐ and anti‐inflammatory genes, as well as genes associated with proliferation and apoptosis (Abizaid & Hougland, 2019; Akalu et al., 2020; Lee et al., 2011; Shi et al., 2017; Zeng et al., 2018; Zheng et al., 2019).

It is also known that GHS‐R1a forms homo‐ and heterodimers with other receptors, which affects the activity of GHS‐R1a (Leung et al., 2007). GHS‐R1a forms a complex with the GHS‐R1b receptor, which is localized on the membranes of the endoplasmic reticulum, which leads to the internalization of GHS‐R1a and a decrease in ligand binding. When GHS‐R1b expression exceeded GHS‐R1a expression, a decrease in GHS‐R1a expression on the cell surface was observed, followed by a decrease in phosphatidylinositol‐specific phospholipase C (PI‐PLC) activity (Leung et al., 2007). GHS‐R1a forms heterodimers with other GPCR receptors (Wellman & Abizaid, 2015). Formation of GHS‐R1a heterodimers with DRD1 (dopamine receptor D1) and DRD1 activation result in noncanonical signaling along the Gαq/11 pathway. It has been suggested that the destruction of these heterodimers may be a mechanism for the development of Alzheimer's disease (Kern et al., 2015; Tian et al., 2019). GHS‐R1a can form dimers with DRD2, as well as with 5HT2c, melanocortin (MC3R, MC4R), somatostatin, and oxytocin receptors (Damian et al., 2018; Schellekens et al., 2013). Activation of 5HT2c within 5HT2c/GHS‐R1a dimers results in inhibition of ghrelin‐induced signaling along the Gαq/11 pathway, while blocking 5HT2c enhances GHS‐R1a signaling and ghrelin‐induced signaling (Schellekens et al., 2015). In vitro analysis shows that GHS‐R1a and MRAP2 form complexes that enhance ghrelin‐induced signaling through Gαq/11 (Srisai et al., 2017). Recent evidence suggests that liver‐expressed antimicrobial peptide 2 (LEAP2), an endogenous ligand of GHS‐R1a, regulates the transmission of intracellular signals from ghrelin (Ge et al., 2018; M’Kadmi et al., 2018; Mani et al., 2019; Wang et al., 2019).

Recently, more and more data have appeared on the so‐called ligand‐independent activity of GHS‐R1a, that is, about the active conformational state of the receptor in the absence of ligands. It is assumed that this activity is important for many physiological processes (Ribeiro et al., 2021).

## 3 CONCLUSIONS

The data obtained on various model objects using molecular genetics and histochemical methods indicate the wide localization of GHS‐R1a in various structures of the brain, which explains the involvement of the ghrelin receptor in many physiological processes. Despite some differences in the nature of the distribution of receptor expression at the periphery, there is great similarity in the distribution of GHS‐R1a in the structures of the brain of model organisms, such as rodents, and primates, and also in humans. Thus, the study of the mechanisms of ghrelin signaling in the rodent brain is justified, given the similarity of the pattern of distribution of GHS‐R1a expression in the brain. The study and understanding of the mechanisms of ghrelin signaling may open new approaches to the pharmacological correction of the pathological conditions of the nervous system in which GHS‐R1a plays an important role.

## COMPLIANCE WITH ETHICAL STANDARDS

This article does not contain any research involving humans or the use of animals as objects.

## CONFLICT OF INTEREST

The authors declare that they have no conflict of interest.

## AUTHOR CONTRIBUTIONS

MIA, SOE, AAL, ERB, and PDS wrote the manuscript.
